# Bridging the Gap: Promoting Physical Activity in College-Aged Students

**DOI:** 10.5888/pcd22.250118

**Published:** 2025-07-10

**Authors:** G. Jack Scroggs, Rebecca A. Battista, Rebecca M. Kappus

**Affiliations:** 1 Appalachian State University, Boone, North Carolina

Physical inactivity is a public health concern and contributes to chronic diseases such as obesity, cardiovascular disease, and diabetes. Evidence supports a strong correlation between physical activity (PA) and long-term health benefits, such as increased longevity and improved quality of life (1,2). Although much research on PA focuses on older adults, college-aged students represent a critical population in a transitional phase where lifelong health behaviors are established (3). However, most of this group fails to meet PA guidelines (3). Despite the well-documented benefits of PA, colleges and universities often lack policies that encourage and support active lifestyles. Unlike K-12 schools, which have state and federal PA policies, higher education institutions lack similar oversight, leaving college-aged students without adequate support for regular PA. Addressing this gap is essential, as changes in PA levels during this stage may have long-term health consequences (4).

This essay explores the importance of examining PA levels in college-aged students from a public health perspective and explores how policy can support active lifestyles in higher education environments. Policy plays a crucial role in shaping health behaviors, and implementing effective PA policies in colleges and universities could foster long-term engagement in PA (5). This essay aims to analyze factors influencing PA behaviors among college-aged students and proposes evidence-based strategies to promote positive behavioral changes.

## College-Aged Students

College-aged students represent a crucial yet neglected population in PA initiatives (3). This group, typically aged 18 to 24 years, experiences a transformative period marked by increased independence, academic stress, shifting social structures, and worsening health behaviors such as poor sleep and nutrition, increased smoking and drinking, and declining mental health (4). PA can help mitigate these challenges by improving overall well-being, easing the transition into adulthood, and reinforcing positive lifelong habits. Research shows that PA levels decline significantly after high school, with many young adults failing to meet recommended guidelines (3,6). Because habits formed during this time persist into adulthood, promoting PA is vital for reducing the long-term burden of chronic diseases such as cardiovascular disease, obesity, and type 2 diabetes (1,2).

Despite these concerns, college-aged students may be overlooked in PA promotion efforts, highlighting the need for more specific and inclusive approaches within this population (4). Although structured PA opportunities such as sports and recreation programs exist, participation is inconsistent, and barriers like academic demands, financial constraints, and shifting social priorities often lead college-aged students to deprioritize PA (4,5). Many also fail to recognize the long-term consequences of sedentary behavior, likely due to a lack of knowledge on the benefits of exercise (6).

## Prevalence of Disease in College-Aged Students

College-aged students are often seen as a generally healthy population, which leads to a limited focus on the part of public health practitioners on their overall health. However, chronic health conditions are apparent within this age group and can significantly affect academic performance, physical well-being, and long-term health outcomes (4,7). Obesity and hypertension, both linked to cardiovascular and metabolic disorders, are increasing among adults aged 18 to 24 years, increasing the likelihood of cardiovascular disease later in life (7). Given these trends, promoting regular PA and decreasing sedentary behavior is important in reducing the risk of chronic disease in this population. Mental health is another growing concern among college-aged students, with 61% reporting moderate to severe psychological distress and 39% having received a clinical diagnosis of a mental health disorder (8). Notably, PA has been shown to reduce symptoms of anxiety and depression while improving cognitive function and academic performance (7).

The burden of cardiovascular risk factors, obesity, and sedentary behavior in this age group suggests a need for behavior-related interventions to help ameliorate poor health outcomes in the future. Since health behaviors are formed early in life (eg, during college years), college campuses are well-positioned to offer health promotion initiatives including increasing student access to preventive health screenings, campus-wide physical activity and nutrition programs, and smoking cessation efforts (6).

## Implementation

To efficiently improve health among college-aged students requires the implementation of comprehensive health promotion models. The National Physical Activity Plan (NPAP) and the Okanagan Charter provide guidance for institutions to improve student health and well-being. The NPAP is a comprehensive set of policies, programs, and initiatives created to increase PA across all demographic segments of the US population. Similarly, the Okanagan Charter encourages colleges and universities to intertwine health into all aspects of campus life and to lead health promotion initiatives at both local and global levels. Incorporating these frameworks creates a foundation for sustainable, evidence-based approaches that can be practically applied to increase PA, support student well-being, and reduce the prevalence of chronic disease on college campuses.

The NPAP provides actionable evidence-based strategies designed for higher education through its Education Sector recommendations (5). Strategy 5 encourages colleges and universities to implement programs and incentives that support physically active lifestyles, while Strategy 6 underscores the importance of policy development (5). Each strategy is followed by tactics and objectives that allow for ease of implementation. One example includes having PA courses as graduation requirements and available as noncredit courses. These courses allow students to be active while learning about the importance of having a physically active lifestyle. Another example would be encouraging recreational centers to have accessible facilities and programs which may include weight rooms, spaces for group personal training, or fitness opportunities. To encourage sustainability, the NPAP also calls for collaboration across campus, such as between health services and recreation staff, to create unified messaging and reinforce a culture that values activity, promotes shared responsibility, and allows for continuous improvement based on student input and institutional data (5).

The Okanagan Charter’s central calls to action are to embed health into every part of campus life and to lead health promotion through university policies and practices. These principles have been effectively implemented at the University of Illinois Urbana-Champaign (9). Following its adoption of the charter, the university developed walkable campus pathways, offered subsidized group fitness sessions, and launched messaging campaigns that encouraged movement throughout the academic day. These changes were built into institutional planning and policies, which helped ensure their long-term impact (9). To address common barriers such as academic stress or lack of motivation, programs must remain flexible, be informed by students, and be culturally inclusive (4). This includes options like peer-led fitness groups, inclusive recreation events, and alternatives to traditional gymnasium settings. Incorporating the NPAP and/or the Okanagan Charter into university settings creates a structured framework to effectively address the health needs of college-aged students and that translates to meaningful clinical improvements. By embedding health into all facets of campus life and promoting active lifestyles, institutions can significantly decrease chronic disease risks and create a healthier, more productive student population (5,9,10).

## Conclusion

College-aged students are in a stage of life where lifelong habits are formed, yet they remain an overlooked demographic segment in PA promotion. The decline in PA levels after high school, along with the increasing prevalence of chronic diseases among college-aged students, highlights the urgent need for targeted interventions within higher education settings (5–7). Unlike the structured PA policies found in K-12 schools, colleges and universities lack the institutional support necessary to encourage active lifestyles. With only 56.9% of college-aged students feeling that their health and well-being are prioritized by their institution, more comprehensive approaches are clearly needed (8). By integrating parts of the NPAP and/or the Okanagan Charter, campuses can take a structured evidence-based approach to embedding health promotion into campus life (9,10).

Policies rooted in these principles, such as campus-wide fitness initiatives, faculty-led PA programs, and incentive-based programs, can create a culture where PA is both accessible and normalized (5). If implemented effectively, these strategies could reshape the future of student health, leading to a generation that is more active, healthier, and better equipped to prevent chronic disease. By prioritizing PA within higher education, campuses have the power to foster lifelong healthy behaviors, reducing health care burdens and improving quality of life well beyond graduation.
